# From elective protocols to emergency reality: rethinking adipose tissue harvesting for stromal vascular fraction applications

**DOI:** 10.1093/stcltm/szag050

**Published:** 2026-07-28

**Authors:** Caroline Nonnarath, Nicolas Serratrice

**Affiliations:** NeuroFAT, Marseille, 13160, France; NeuroFAT, Marseille, 13160, France; Institut Marseille Rachis, Vert coteau Private Hospital, Marseille, 13012, France

**Keywords:** adipose tissue harvesting, stromal vascular fraction, emergency surgery, acute care, enzymatic digestion, mechanical isolation, systemic inflammation, cell viability, regenerative medicine, neurosurgery, point-of-care therapy

## Abstract

**Background:**

Adipose tissue is a readily accessible source of stromal vascular fraction (SVF) and adipose-derived stem/stromal cells, widely used in regenerative medicine. Current harvesting and processing standards are derived almost exclusively from elective surgical settings performed under controlled physiological conditions. However, expanding applications in trauma and neurosurgery increasingly require adipose tissue procurement in emergency settings, where systemic inflammation, hypoperfusion, and time constraints may alter tissue biology. The biological impact of emergency harvesting remains insufficiently characterized, yet this knowledge is critical for optimizing SVF-based regenerative medicine strategies in emergency surgical environments.

**Objectives:**

To evaluate how emergency adipose procurement may influence SVF quality, cellular yield, viability, and translational feasibility, and to propose an acute-care–adapted framework for clinical integration.

**Methods:**

A comprehensive search of PubMed, EMBASE, and Scopus (inception–January 2026) identified studies addressing harvesting techniques, donor-site variability, enzymatic vs mechanical isolation, time-to-processing effects, cryopreservation, contamination risk, and clinical outcomes. Data were synthesized narratively with emphasis on variables relevant to emergency settings.

**Results:**

Harvesting parameters—including cannula diameter and negative pressure—influence total nucleated cell yield and viability. Enzymatic digestion increases cell recovery by approximately 1.5- to 3-fold compared with mechanical fractionation but requires laboratory infrastructure and a longer processing time. Mechanical techniques enable rapid point-of-care application yet produce stromal vascular tissue with greater heterogeneity. Processing delays reduce viable cell counts. Emergency physiology introduces additional upstream modifiers, including cytokine activation and microvascular perturbation, that remain largely unstudied in SVF research.

**Conclusions:**

Emergency adipose harvesting challenges elective-based assumptions underlying current SVF standards. Context-specific comparative studies and standardized emergency-adapted protocols are required to ensure biological robustness and clinical safety in acute surgical environments.

## Graphical Abstract

**Graphical Abstract:**
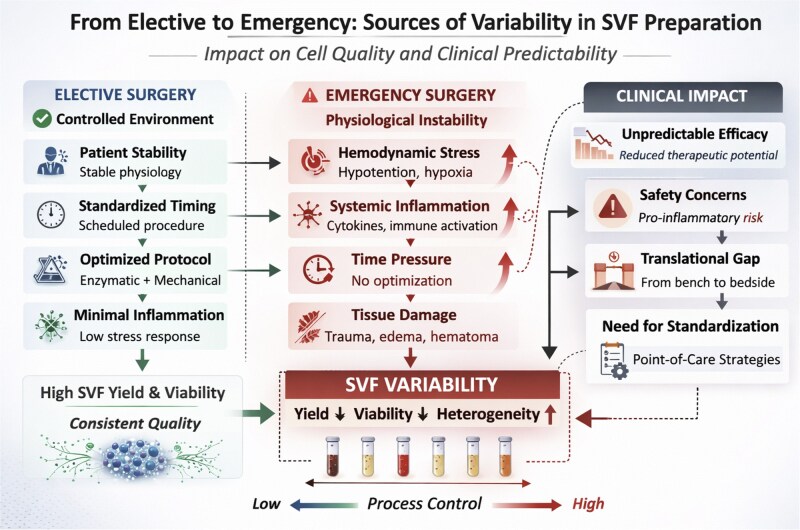

Significance statement Adipose-derived therapies are increasingly used beyond elective procedures, including in emergency and high-acuity surgical settings. This review highlights that adipose tissue is a context-sensitive biological source whose regenerative potential may be influenced by systemic stress and procedural constraints. By identifying key sources of variability and proposing an acute-care–adapted framework, this work supports safer and more consistent clinical translation of stromal vascular fraction therapies in urgent conditions.

## Introduction

Adipose tissue has emerged as one of the most accessible and clinically versatile sources of regenerative cellular products. The stromal vascular fraction (SVF), a heterogeneous cell population obtained from lipoaspirate, contains adipose-derived stem/stromal cells (ADSCs), endothelial progenitors, pericytes, immune cells, and vascular-associated elements that collectively contribute to tissue repair, angiogenesis, and immunomodulation.^1–3^ Because of its relative abundance, ease of harvest, and autologous availability, SVF has been increasingly integrated into reconstructive surgery, fat grafting procedures, and translational regenerative applications.^4^^,^^5^

Current technical standards for adipose tissue procurement and SVF isolation have largely been established within elective surgical settings. In these controlled environments, harvesting techniques—such as syringe aspiration, vacuum-assisted liposuction, or water-jet assisted lipoplasty—are optimized to preserve cellular integrity.^6–8^ Processing protocols, whether enzymatic digestion or mechanical fractionation, are typically performed under standardized laboratory or quasi-laboratory conditions.^9–12^ These optimized workflows underpin most published data regarding SVF yield, viability, immunophenotype, and functional capacity.^6^^,^^11^^,^^13^

Multiple studies have demonstrated that harvesting modality, cannula diameter, applied negative pressure, and donor-site selection significantly influence total nucleated cell yield and cellular viability.^6^^,^^8^^,^^14^ Furthermore, the choice between enzymatic digestion and mechanical isolation determines not only cell recovery rates but also the cellular composition of the final product and its regulatory classification.^10^^,^^11^^,^^15–17^ Enzymatic isolation generally provides higher cell yields but requires extended processing time and laboratory infrastructure, whereas mechanical techniques enable point-of-care application at the cost of compositional heterogeneity.^15–17^

The safety profile of autologous SVF therapies has been reported as favorable across multiple clinical indications.^5^^,^^18^ However, these safety assessments are primarily derived from elective contexts in which harvesting and processing are performed under standardized conditions. Cryopreservation and storage studies further demonstrate that procedural handling directly impacts viable cell counts and functional potency.^13^

The growing interest in point-of-care regenerative interventions is progressively extending adipose-derived therapies beyond elective procedures. Acute trauma surgery, reconstructive emergencies, and selected neurosurgical applications increasingly consider SVF-based approaches within time-sensitive environments.^3^^,^^17^ In such settings, deviations from elective standards—including expedited harvesting, constrained sterility pathways, delayed or simplified processing, and limited laboratory infrastructure—may introduce biological variability that has not been systematically characterized (Table 1). Relevant clinical scenarios include acute trauma reconstruction, ischemic tissue rescue, and selected neurosurgical interventions where rapid regenerative support may be beneficial.

**Table 1.** Emergency vs elective differences.

Parameter	Elective	Emergency
**Time-to-processing**	Flexible	≤60 min target
**Processing type**	Enzymatic (standard)	Mechanical (preferred); enzymatic possible if infrastructure available
**Infrastructure**	Lab-supported	OR-based
**Consent**	Standard	Emergency-adapted
**Documentation**	Standard	Rapid+traceability

This represents a critical translational gap. While extensive literature addresses optimization of adipose harvesting and SVF isolation under controlled conditions,^6^^,^^10^^,^^15^ the biological robustness of these protocols when applied in emergency surgical contexts remains largely unexplored. These combined biological and operational constraints create a translational uncertainty that remains insufficiently explored.

Early and recent studies confirm that enzymatic digestion improves SVF yield and fat graft maintenance compared with mechanical approaches, while adipose tissue also acts as a bioactive scaffold.^19–21^

The objective of this scoping review is, therefore, to analyze existing evidence on adipose tissue harvesting and SVF isolation through the lens of emergency applicability. Specifically, we aim to (1) identify biological variables susceptible to alteration in acute procurement settings, (2) assess the feasibility and implications of mechanical vs enzymatic isolation under time constraints, (3) evaluate safety and regulatory considerations, and (4) propose a structured framework for integrating adipose tissue harvesting into emergency surgical workflows.

By systematically addressing this overlooked dimension of regenerative medicine, this work seeks to expand current procurement paradigms beyond elective settings and to establish a foundation for acute-care–adapted regenerative strategies.

## Methods

### Study design

This study was designed as a scoping review aimed at mapping existing evidence regarding adipose tissue harvesting techniques and SVF isolation procedures, with a specific translational focus on their applicability to emergency surgical settings. The review was conducted in accordance with the methodological framework for scoping reviews and is reported following PRISMA-ScR recommendations. A total of 312 records were identified. After removal of duplicates, 248 were screened. Seventy-four full-text articles were assessed, and 42 were included.

### Search strategy

A comprehensive literature search was performed in PubMed/MEDLINE, EMBASE, and Scopus from database inception through January 2026. The search strategy combined controlled vocabulary (MeSH/Emtree terms) and free-text keywords related to adipose tissue harvesting, SVF isolation, regenerative applications, and time-sensitive or acute surgical contexts. The core search string used in PubMed was: (“Adipose Tissue” [MeSH] OR “fat grafting” OR “lipoaspirate” OR “adipose-derived stem cells” OR “stromal vascular fraction”) AND (“harvest*” OR “liposuction” OR “tissue procurement” OR “donor site”) AND (“mechanical isolation” OR “enzymatic digestion” OR “processing” OR “centrifugation”) AND (“emergency” OR “acute” OR “trauma” OR “time-to-processing” OR “point-of-care” OR “intraoperative”). Additional targeted searches were performed for cryopreservation, contamination risk, donor-site variability, regulatory considerations for minimally manipulated tissues, and neurosurgical and trauma applications. Reference lists of included studies were manually screened to identify additional relevant publications.

### Eligibility criteria

Inclusion Criteria: Studies were included if they:

- Investigated adipose tissue harvesting techniques (liposuction, excision, water-jet, syringe aspiration);
- Reported outcomes related to SVF or ADSC yield, viability, phenotypic characterization, or functional assays;
- Compared mechanical vs enzymatic isolation methods;
- Examined donor-site variability;
- Assessed processing delay, storage conditions, cryopreservation, or contamination risk;
- Reported clinical applications of autologous SVF or cell-assisted fat grafting;
- Were published in peer-reviewed journals in English.

Both preclinical and clinical studies were considered to ensure comprehensive translational coverage.

Exclusion Criteria: Studies were excluded if they:

- Focused solely on cultured-expanded ADSCs without describing primary SVF isolation;
- Investigated non-adipose stem cell sources;
- Were conference abstracts without full text;
- Lacked methodological detail on harvesting or processing;
- Were purely theoretical without experimental or clinical data.

### Study selection

Two independent reviewers screened titles and abstracts for eligibility. Full texts were assessed when relevance was unclear. Discrepancies were resolved by consensus discussion.

### Data extraction

For each included study, the following variables were extracted:

- Harvesting technique (liposuction type, cannula diameter, negative pressure);
- Donor site;
- Processing method (enzymatic vs mechanical);
- Time from harvest to processing;
- Storage conditions (temperature, cryopreservation);
- Total nucleated cell count;
- Cell viability (Trypan Blue, Annexin V, flow cytometry);
- Mesenchymal stromal cell (MSC) immunophenotype (CD73, CD90, CD105);
- Colony-forming unit–fibroblast (CFU-F) frequency;
- Functional assays (angiogenesis, differentiation capacity);
- Reported complications or contamination events;
- Regulatory classification (minimally manipulated vs substantially manipulated).

### Data synthesis

Due to heterogeneity across studies, a quantitative meta-analysis was not feasible. Findings were, therefore, synthesized narratively, focusing on biological robustness, feasibility in acute settings, safety, and regulatory considerations.

### Conceptual framework development

Based on the synthesized evidence, a translational framework was constructed to identify critical variables influencing SVF quality in emergency settings. This framework informed the development of practical recommendations and a proposed acute-care–adapted harvesting pathway.

## Review

### Techniques of adipose tissue harvesting

Liposuction remains the most widely employed technique for adipose tissue procurement in regenerative medicine. Variations in harvesting parameters—including negative pressure, cannula diameter, aspiration modality (manual syringe vs vacuum-assisted systems), and water-jet assistance—have been shown to influence adipocyte integrity and SVF yield. Lower negative pressure systems, particularly syringe-based aspiration, are generally associated with improved cell viability compared with high-vacuum devices, likely due to reduced mechanical shear stress.^6^^,^^8^ Cannula diameter further modulates tissue trauma: smaller cannulas may increase shear forces and disrupt adipocyte membranes, whereas larger diameters may better preserve stromal architecture at the expense of greater tissue displacement. Water-jet–assisted lipoplasty has been proposed as a gentler alternative, with preliminary evidence suggesting enhanced preservation of stem cell viability and adipose microstructure.^7^

In contrast, direct surgical excision yields intact adipose lobules and theoretically preserves stromal integrity; however, it requires additional operative exposure and may prolong ischemia time before processing. Comparative studies indicate that excised adipose tissue can provide viable SVF populations when rapidly processed, yet prolonged ischemic intervals may negatively affect cellular viability and functional capacity.^6^ In emergency contexts, peroperative harvesting performed during trauma or reconstructive procedures may represent a pragmatic solution. Nevertheless, the inflammatory milieu, local tissue hypoperfusion, electrocautery exposure, and variable handling conditions inherent to acute surgery may introduce biological variability that has not been systematically quantified.

### Donor-site variability

Anatomical origin significantly influences adipose-derived stem cell frequency and SVF yield. Abdominal adipose tissue is most frequently utilized due to its accessibility and consistent cellular yield.^14^ Comparative analyses have demonstrated that thigh and flank depots may exhibit differences in progenitor frequency and stromal composition, potentially reflecting depot-specific vascular density and metabolic characteristics.^6^ While elective procedures allow deliberate donor-site optimization, emergency harvesting may be dictated by surgical positioning, wound location, or immediate accessibility rather than biological superiority. This pragmatic selection may inadvertently influence total nucleated cell recovery and stromal composition. Furthermore, trauma-associated systemic inflammation may differentially impact adipose depots, a variable that remains insufficiently explored.

### Processing and isolation strategies

The method of SVF isolation critically determines cellular yield, phenotypic composition, and regulatory classification. Enzymatic digestion, typically employing collagenase-based protocols, remains the reference standard for maximal recovery of total nucleated cells and mesenchymal stromal populations.^11^^,^^15^ This approach enables relatively homogeneous cell suspensions and high recovery efficiency but requires controlled laboratory infrastructure, incubation periods of 30-90 min, multiple washing steps, and regulatory oversight due to substantial manipulation.

Mechanical fractionation techniques—including centrifugation, filtration, emulsification, and nanofat processing—offer rapid, enzyme-free alternatives that can be performed at the point-of-care.^10^^,^^16^ These techniques yield a more heterogeneous product (tSVF) containing extracellular matrix components.^17^ Although mechanical isolation may be biologically less standardized than enzymatic digestion, it is considerably more compatible with emergency surgical environments where laboratory infrastructure and processing time are limited.

Centrifugation parameters further influence cell viability. Excessive centrifugal force may damage fragile adipocytes and stromal cells, whereas insufficient force may reduce separation efficiency.^4^ Simple decantation preserves structural integrity but yields lower cell concentration, and filtration-based systems may provide reproducible enrichment at the expense of additional equipment requirements. The feasibility of each method must, therefore, be evaluated within the operational constraints of acute surgical settings.

### Commercial and point-of-care SVF isolation technologies

In recent years, several commercial systems have been developed to standardize SVF isolation and facilitate point-of-care application. These technologies include both enzymatic and mechanical closed-system devices designed to improve reproducibility, sterility, and workflow integration. Enzymatic platforms typically aim to automate digestion, washing, and cell recovery under controlled conditions, whereas mechanical systems rely on filtration, centrifugation, or emulsification processes without the use of enzymes.

Such systems may reduce operator-dependent variability and improve procedural standardization. However, their applicability in emergency settings remains dependent on availability, processing time, regulatory classification, and integration into acute surgical workflows. While closed-system devices may enhance safety and traceability, their logistical constraints may limit their use in high-acuity environments. Therefore, selection of these technologies in emergency contexts must balance standardization benefits against operational feasibility.

### Impact of time and logistics chain

Time to processing is a critical determinant of cellular viability and functional potency. Experimental evidence demonstrates that prolonged intervals between tissue procurement and SVF isolation reduce viable cell counts and may impair stem cell functionality.^13^ Temperature control during transport also influences survival; refrigeration may preserve viability for short intervals, whereas uncontrolled room-temperature exposure may accelerate apoptotic pathways.

Cryopreservation studies indicate that although total nucleated cell counts may decrease following freezing and thawing, stem cell potency can be preserved when appropriate protocols are applied.^13^ However, cryopreservation is rarely feasible in emergency operative workflows. In acute contexts, logistical challenges—including operating room turnover, interdepartmental transport, and limited access to sterile processing facilities—may extend time-to-processing beyond optimal windows. These delays introduce additional biological variability and may increase contamination risk, particularly when sterility pathways are modified under time pressure.

### Measurable indicators of tissue and SVF quality

Objective assessment of adipose tissue quality in emergency settings requires standardized quantitative metrics. Total nucleated cell count and viability assays, including Trypan Blue exclusion or Annexin V flow cytometry, provide fundamental indicators of cellular integrity. Immunophenotypic characterization of mesenchymal stromal populations through expression of CD73, CD90, and CD105 further defines regenerative potential.^11^^,^^15^ CFU-F assays quantify clonogenic capacity, while *in vitro* angiogenic or differentiation assays evaluate functional potency.

Microbiological contamination screening is essential, particularly in acute surgical environments where sterility may be compromised. Additionally, profiling inflammatory cytokines within processed adipose tissue may offer insight into trauma-induced alterations in cellular behavior. Collectively, these parameters provide a framework for comparing elective and emergency procurement conditions and for identifying biologically meaningful deviations.

### Integration into the clinical workflow

In elective settings, adipose harvesting and SVF processing are typically embedded within structured surgical pathways that allow controlled timing, personnel allocation, and laboratory support. In contrast, emergency procurement must integrate into high-acuity operative workflows, such as trauma or neurosurgical interventions, where time constraints and clinical priorities differ substantially. Harvesting may occur intraoperatively, with processing performed either in adjacent facilities or using bedside mechanical systems.

Regulatory classification plays a pivotal role in workflow feasibility. Mechanical fractionation may qualify as minimal manipulation depending on jurisdiction, whereas enzymatic digestion is frequently categorized as substantial manipulation requiring good manufacturing practice (GMP) facilities.^17^ Multidisciplinary coordination among surgeons, anesthesiologists, and cell-processing personnel is, therefore, essential to maintain compliance, traceability, and sterility.

### Risks and safety considerations

Autologous SVF therapies have demonstrated an overall favorable safety profile across multiple clinical indications.^5^^,^^17^ Nevertheless, emergency contexts may increase susceptibility to procedural complications. Infection risk may rise when sterility pathways are shortened or when processing occurs outside dedicated facilities. Hemorrhagic complications may occur if harvesting is performed in hemodynamically unstable patients. Furthermore, obtaining informed consent in urgent scenarios introduces ethical complexity.

The intersection of biological variability and procedural urgency underscores the necessity for clearly defined safety protocols adapted to acute environments.

### Why enzymatic isolation may be justified in emergency settings despite operational constraints

Although mechanical techniques are often preferred in acute settings due to their simplicity, enzymatic isolation may be justified when biological consistency is prioritized. Enzymatic digestion provides higher cell yield and a more homogeneous SVF population compared with mechanical approaches.^11^^,^^15^ This may be advantageous in severe clinical conditions where regenerative potential is critical. By fully dissociating adipose tissue, enzymatic processing may also reduce variability introduced during harvesting under unstable physiological conditions. In addition, enzymatically isolated SVF enables more standardized assessment of viability, phenotype, and functional potential, supporting more reliable quality control and comparative research. However, enzymatic isolation requires dedicated infrastructure, longer processing time, and regulatory compliance,^17^ limiting its feasibility in many emergency settings. Therefore, its use should remain indication-driven and restricted to centers with appropriate capabilities. In the absence of emergency-specific comparative data, current practice continues to rely on extrapolation from elective conditions.

### When mechanical isolation may be preferable in emergency contexts

Mechanical isolation may be preferable in emergency settings where rapid deployment and operational simplicity are critical. In high-acuity situations, processing must be performed within minutes, making enzymatic digestion impractical. Mechanical techniques can be performed at the point-of-care with minimal infrastructure, reducing dependency on laboratory facilities and simplifying regulatory constraints. This approach facilitates integration into urgent surgical workflows. Although associated with lower cell yield and greater heterogeneity compared with enzymatic isolation,^11^^,^^15^ mechanically derived SVF may provide sufficient regenerative support in time-sensitive conditions. Residual extracellular matrix components may also contribute to structural support and cell retention. In addition, reduced handling steps may limit contamination risk and operator-dependent variability in non-laboratory environments. Overall, mechanical isolation represents a pragmatic strategy when immediate treatment is required or when infrastructure and regulatory conditions do not allow enzymatic processing. However, comparative data in emergency contexts remain lacking.

### Knowledge gaps and future research directions

Despite extensive literature on adipose harvesting and SVF optimization in elective settings, direct comparisons between elective and emergency procurement are lacking. The influence of ischemia time, systemic inflammatory response, and intraoperative stressors on SVF composition remains insufficiently characterized. Prospective comparative studies are urgently needed to quantify differences in cell yield, viability, phenotype, and contamination risk between contexts.

A pilot comparative study evaluating elective vs emergency harvesting under standardized measurement criteria would provide essential translational data and inform the development of acute-care–adapted protocols.

### Limitations

This review has several limitations that should be acknowledged. First, the available literature on adipose tissue harvesting and SVF isolation is overwhelmingly derived from elective surgical settings, limiting the direct extrapolation of findings to emergency contexts. No study to date has specifically compared elective vs emergency adipose procurement under standardized conditions, which constrains the strength of translational inferences drawn in this review. Second, methodological heterogeneity across studies—including differences in harvesting techniques, processing protocols, outcome reporting, and viability assessment methods—precluded quantitative synthesis and limited cross-study comparability. Third, much experimental data originate from preclinical or laboratory-based investigations conducted under controlled conditions that may not accurately reflect the biological and logistical variability encountered in acute surgical environments. Regulatory considerations also vary across jurisdictions, potentially affecting the generalizability of workflow recommendations. Finally, the scoping design of this review, while appropriate for mapping existing evidence and identifying knowledge gaps, does not allow for formal risk-of-bias assessment or meta-analytic evaluation. These limitations underscore the need for prospective, context-specific comparative studies to validate the proposed framework and to establish evidence-based standards for emergency adipose tissue procurement.

### Proposed standard operating procedure for emergency adipose tissue harvesting and point-of-care enzymatic SVF isolation

**Figure 1.**
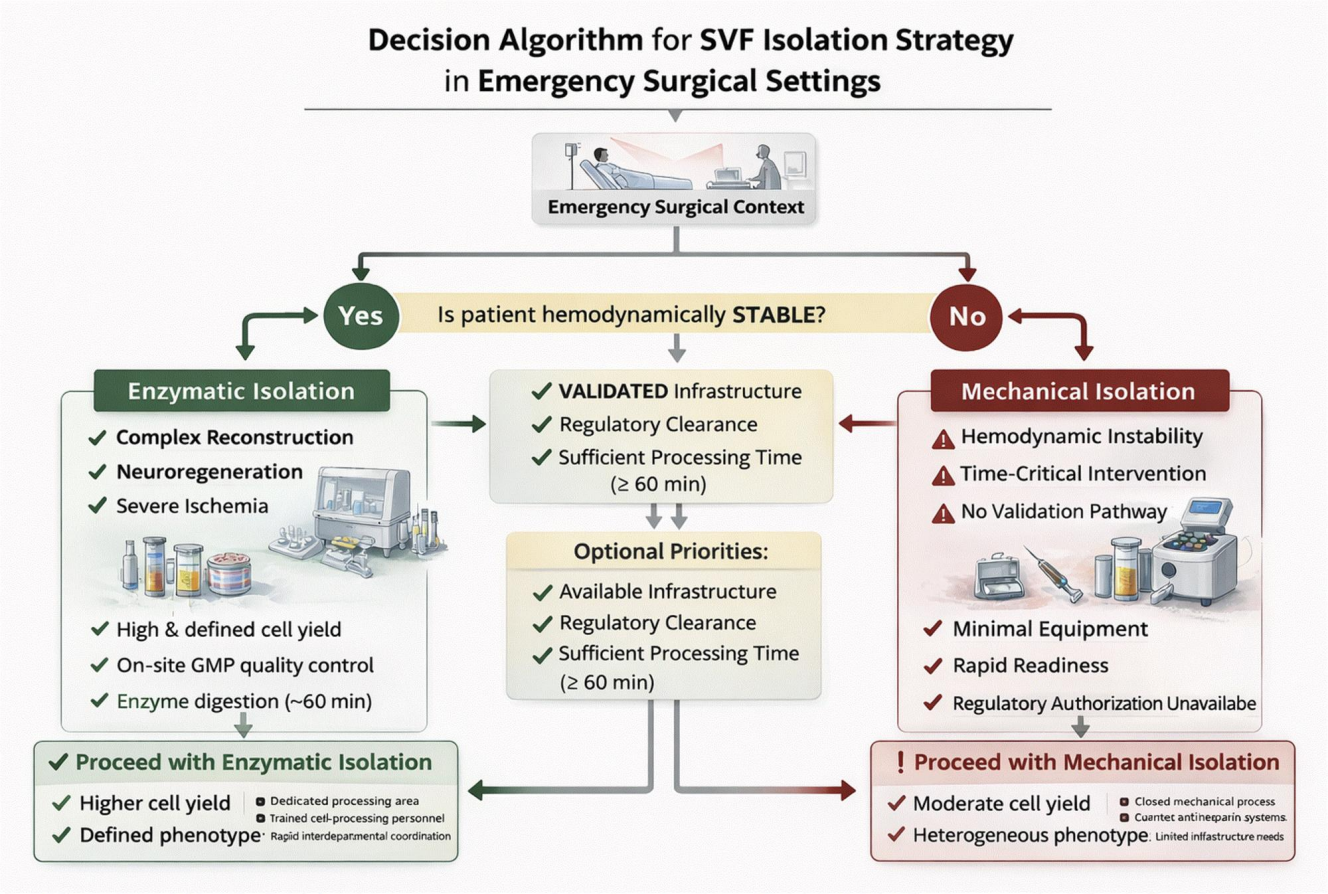
Flowchart showing decision-making for SVF isolation strategy in emergency surgical settings based on clinical urgency and infrastructure. Decision algorithm for SVF isolation strategy in emergency surgical settings. This table illustrates a structured decision pathway for selecting an SVF isolation strategy in emergency surgical environments. The algorithm integrates clinical urgency, institutional infrastructure, biological objectives, and regulatory considerations. The decision process begins with an assessment of patient hemodynamic stability and procedural urgency. In unstable or time-critical scenarios requiring immediate regenerative support, mechanical isolation is favored due to its rapid execution and point-of-care feasibility. In hemodynamically stable patients, further evaluation of institutional capacity is performed. If a validated cell-processing facility with appropriate regulatory authorization for enzymatic digestion is available and accessible within a clinically acceptable timeframe (typically ≥60 min), enzymatic isolation may be considered. This approach is particularly justified when high cellular yield and defined phenotypic composition are critical for therapeutic objectives, such as extensive tissue reconstruction, severe ischemic injury, or neuroregenerative applications. In contrast, when laboratory infrastructure is unavailable, regulatory clearance is lacking, or operative workflow constraints limit processing time, mechanical fractionation is recommended. Although associated with lower total nucleated cell yield and greater compositional heterogeneity, mechanical isolation offers reduced logistical complexity, minimal infrastructure dependence, and immediate intraoperative deployment. The algorithm emphasizes that the selection of an isolation strategy in emergency contexts should be indication-driven and institution-specific, balancing biological optimization against operational feasibility and safety. Prospective comparative studies are required to validate outcome differences between strategies under acute conditions.

In selected emergency settings, enzymatic SVF isolation may be performed using an accelerated but controlled workflow in institutions with appropriate infrastructure and regulatory authorization (Table 2 and Figure 1).

**Table 2.** Decision algorithm for SVF isolation strategy in emergency surgical settings.

Clinical/Operational variable	Enzymatic isolation preferred	Mechanical isolation preferred
**Hemodynamic stability**	Stable patient	Unstable/time-critical
**Available infrastructure**	On-site GMP-compliant or validated cell-processing facility	No laboratory access/OR-only setting
**Time available for processing**	≥60-90 min feasible	<30 min required
**Regulatory authorization**	Approved enzymatic manipulation pathway	No authorization for substantial manipulation
**Required cell yield**	High cell number critical (large defect, severe ischemia)	Moderate cell number acceptable
**Need for phenotypic precision**	Defined cellular composition required	Heterogeneous stromal tissue acceptable
**Indication type**	Complex reconstruction, neuroregeneration, ischemic tissue rescue	Adjunctive regenerative support
**Staff expertise**	Trained cell-processing personnel available	Only surgical team available
**Contamination risk tolerance**	Controlled laboratory sterility available	Prefer minimal handling steps
**Institutional readiness**	Pre-established emergency enzymatic protocol	No emergency cell-processing protocol

Before harvesting, the indication should be confirmed, patient stability assessed, and contraindications excluded. An emergency-adapted consent process and predefined coordination between surgical and cell-processing teams are essential to minimize time-to-processing.

Donor-site selection should prioritize accessibility and sterility, with preference for abdominal adipose tissue. Harvesting should be performed using low-negative-pressure aspiration with 3-4 mm blunt cannulas to reduce mechanical trauma. The time of harvest must be documented.

Following procurement, the lipoaspirate should be transferred in a sterile closed system for rapid processing, ideally within 60 min. Short-term storage at 4 °C may be considered if necessary.

Enzymatic isolation should be conducted under sterile conditions using validated protocols, including washing, collagenase digestion, centrifugation, and resuspension of the SVF for immediate use.

Basic quality control measures, including cell count and viability assessment, should be performed when feasible. Sterility sampling and full traceability of processing steps are recommended.

The final product should be administered promptly using a closed sterile pathway. Monitoring for adverse events is required.

This emergency-adapted approach prioritizes biological consistency while acknowledging increased logistical and regulatory constraints. Its implementation should remain indication-driven and institution-specific. Prospective studies are needed to validate its safety and efficacy in acute settings.

## Discussion

The expansion of adipose-derived regenerative strategies into trauma and high-acuity surgical environments challenges a fundamental assumption underlying current translational practice: that SVF quality is independent of physiological context.^22^ Existing optimization data have been generated almost exclusively under elective, hemodynamically stable conditions.^6^^,^^11^^,^^15^ However, SVF is not a fixed product; it is a biologic output shaped by systemic, microvascular, and inflammatory inputs at the time of procurement.

### Emergency physiology as a biological modifier of SVF

**Figure 2.**
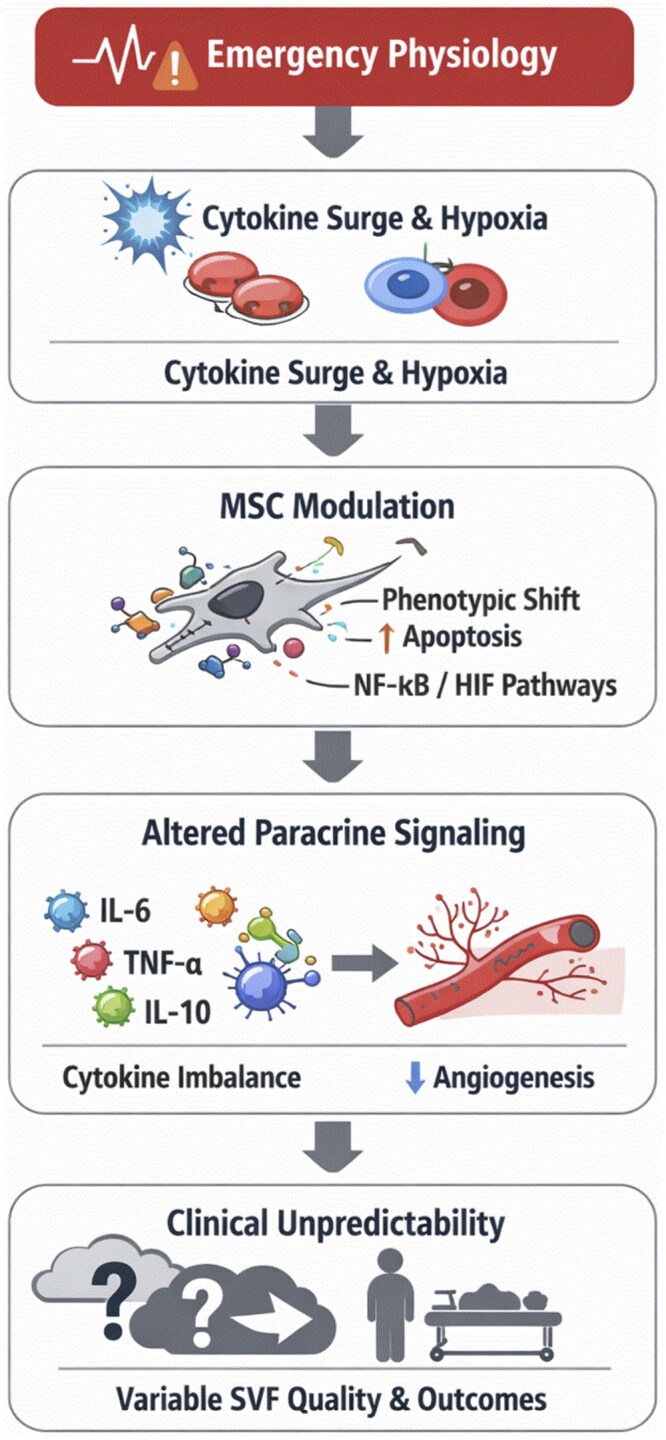
Conceptual diagram illustrating how emergency physiological stress may influence SVF composition and clinical outcomes. This conceptual model illustrates how acute surgical physiology may influence SVF biological composition and downstream clinical performance. Emergency contexts are characterized by systemic inflammatory activation, catecholamine surges, endothelial perturbation, and transient microvascular hypoperfusion. These upstream stressors may induce cytokine elevation and tissue hypoxia within adipose depots prior to harvesting. Inflammatory and hypoxic signaling pathways—including NF-κB–mediated inflammatory cascades and HIF-dependent transcriptional responses—may modulate MSC phenotype, apoptosis susceptibility, and secretory behavior. Such modulation can lead to altered paracrine signaling profiles, including shifts in pro- and anti-inflammatory cytokine balance and changes in angiogenic potential. Collectively, these context-dependent biological modifications may contribute to variability in SVF cellular composition, functional potency, and ultimately regenerative outcomes. The model underscores the need for elective-vs-emergency comparative profiling to determine whether SVF derived from acute settings remains biologically equivalent to that obtained under elective conditions.

Acute trauma and surgical stress are characterized by rapid systemic inflammatory activation, catecholamine surge, endothelial perturbation, and transient microvascular hypoperfusion (Figure 2). Adipose tissue is an immunometabolic organ enriched in mesenchymal stromal cells, endothelial progenitors, pericytes, and immune populations.^1^^,^^2^^,^^23^^,^^24^ These cell populations are known to respond dynamically to inflammatory and hypoxic stimuli.

Experimental evidence indicates that mesenchymal stromal cells exposed to inflammatory cytokines may undergo phenotypic modulation, including altered paracrine signaling, shifts in immunomodulatory function, and changes in proliferative capacity.^11^^,^^15^ Hypoxia-inducible signaling pathways (e.g., HIF-mediated transcriptional responses) and NF-κB–dependent inflammatory cascades may theoretically alter SVF composition prior to isolation. In emergency settings, such upstream activation could modify the following:

- Relative proportion of immune subsets;
- Endothelial cell activation status;
- MSC immunomodulatory phenotype;
- Apoptotic susceptibility.

No study has directly compared inflammatory or endothelial profiles between elective and emergency SVF. This absence of context-specific cellular characterization represents a major translational gap.

### From procedural variability to biological variability

Elective workflows optimize harvesting mechanics, donor-site selection, and time-to-processing.^6^^,^^14^ In contrast, emergency procurement introduces variability at multiple levels:

- Hemodynamic instability altering tissue perfusion;
- Edema modifying mechanical resistance;
- Expedited harvesting increasing shear stress;
- Delays extending ischemia time.

Time to processing has been shown to influence viable cell counts.^13^ When combined with upstream inflammatory activation, even modest procedural deviations may amplify biological variability.

Thus, emergency procurement may not simply reduce cellular yield but may also alter functional phenotype. Quantitative differences in total nucleated cell count (1.5-3-fold variation between enzymatic and mechanical techniques) may be less critical than qualitative shifts in immunophenotype or secretory profiles.

### Isolation strategy as a modulator of translational robustness

In emergency contexts where biological variability is increased, the choice of isolation strategy may further influence downstream consistency. Enzymatic digestion provides higher total nucleated cell counts and yields a more homogeneous cellular suspension, which may help reduce compositional variability under acute conditions. In contrast, mechanical isolation retains extracellular matrix components and cellular aggregates, resulting in a more heterogeneous product.

While both approaches remain clinically relevant, their respective advantages may be amplified in emergency settings where upstream variability is already present. However, no comparative dataset currently evaluates enzymatic vs mechanical SVF derived specifically from emergency conditions. As a result, current translational decision-making still relies largely on extrapolation from elective data.

### Toward a context-sensitive translational framework

Future investigations must move beyond yield and viability toward multidimensional characterization (Table 3). A minimum comparative framework should include the following:

**Table 3.** Proposed biomarker framework for elective vs emergency SVF characterization.

Domain	Parameter	Elective baseline (expected)	Emergency hypothesized shift	Biological rationale	Measurement method
**Cell yield**	Total nucleated cell count (TNC)	Stable, protocol-dependent	↓ or variable	Ischemia, hypoperfusion, harvesting stress	Automated cell counter
	SVF per mL lipoaspirate	Predictable range	Increased variability	Microvascular instability	Cell count normalization
**Viability**	Trypan Blue exclusion	>85%-90% typical	↓	Ischemia-related apoptosis	Trypan Blue
	Annexin V/PI	Low apoptotic fraction	↑ early apoptosis	Hypoperfusion, oxidative stress	Flow cytometry
**MSC phenotype**	CD73+/CD90+/CD105+	Stable MSC fraction	Possibly stable or mildly ↓	Stress-induced phenotype modulation	Flow cytometry
	CFU-F frequency	Consistent clonogenicity	↓	Cytokine-induced functional shift	Colony assay
**Immune composition**	CD45+ total leukocytes	Baseline fraction	↑	Systemic inflammatory activation	Flow cytometry
	CD14+ monocytes/macrophages	Baseline	↑	Trauma cytokine recruitment	Flow cytometry
	CD3+ T cells	Low baseline	↑	Inflammatory mobilization	Flow cytometry
**Endothelial fraction**	CD31+/CD34+ cells	Depot-dependent	Variable (↑ activation or ↓ viability)	Endothelial stress, microvascular perturbation	Flow cytometry
	Endothelial activation markers (e.g., ICAM-1)	Low baseline	↑	Cytokine-induced activation	Immunostaining/flow
**Inflammatory profile**	IL-6	Low baseline	↑	Acute systemic inflammation	ELISA
	TNF-α	Minimal	↑	Trauma response	ELISA
	IL-10	Baseline	Variable ↑	Compensatory anti-inflammatory phase	ELISA
**Hypoxia/Stress response**	HIF-1α expression	Baseline	↑	Tissue hypoperfusion	qPCR/Western blot
	ROS markers	Low	↑	Oxidative stress	Fluorescent probes
**Functional potency**	In vitro angiogenesis	Stable	Variable	Endothelial/MSC modulation	Tube formation assay
	Secretome profile	Baseline pattern	Shifted cytokine balance	Paracrine alteration	Multiplex assay

- Total nucleated cell count;
- Viability (Annexin V/PI);
- MSC phenotype (CD73/CD90/CD105);
- Immune subset profiling (CD45+, CD14+, CD3+);
- Endothelial markers (CD31, CD34);
- Inflammatory cytokine quantification (IL-6, TNF-α, IL-10);
- Functional assays (angiogenesis, CFU-F).

Only through systematic elective-vs-emergency profiling can the field determine whether emergency-derived SVF remains biologically equivalent—or functionally distinct.

The extension of adipose-derived regenerative therapies into high-acuity settings, therefore, demands a conceptual shift: SVF must be regarded as a context-sensitive biologic product rather than a universally reproducible autologous resource. Bridging this gap is essential to preserve translational integrity as regenerative medicine expands into acute care.

### Clinical implications and conceptual reframing

From a clinical perspective, emergency adipose harvesting should not be presumed biologically equivalent to elective procurement. Awareness of procedural and physiological variability is critical for surgical teams integrating SVF-based therapies into high-acuity workflows. Structured decision algorithms and standardized documentation of harvest conditions may mitigate variability and improve reproducibility.

More broadly, adipose tissue should no longer be conceptualized solely as a “convenience” autologous source of regenerative cells. As applications expand into emergency and neurosurgical contexts,^17^ it must instead be regarded as a context-sensitive biologic product whose quality is influenced by systemic physiology, harvesting mechanics, processing methodology, and regulatory environment. Recognizing this complexity is essential to ensure that regenerative interventions maintain biological integrity across surgical contexts.

## Conclusions

Adipose tissue harvesting has been widely adopted as a cornerstone of regenerative medicine, yet the standards governing its procurement and processing remain fundamentally rooted in elective surgical paradigms. As SVF-based therapies expand into trauma, reconstructive emergencies, and neurosurgical applications, the assumption of biological equivalence between elective and emergency procurement can no longer remain implicit.

Emergency adipose harvesting is technically feasible, but it occurs within a distinct physiological and operational landscape. Systemic inflammatory activation, transient hypoperfusion, harvesting under time pressure, constrained sterility pathways, and limited infrastructure collectively introduce sources of biological and procedural variability that have not been systematically quantified. While elective data demonstrate that harvesting mechanics, isolation strategy, and processing delays significantly influence SVF yield and viability, the impact of acute-care-specific modifiers remains largely unexplored.

This gap represents a critical translational blind spot. Without context-specific evidence, regenerative therapies deployed in high-acuity environments risk relying on assumptions derived from physiologically stable cohorts. Moving forward, the field must transition from extrapolation to validation. Prospective elective-vs-emergency comparative studies incorporating quantitative cellular metrics, inflammatory profiling, and standardized workflow documentation are urgently required.

Establishing acute-care–adapted harvesting protocols, embedded quality-control checkpoints, and clearly defined regulatory pathways will be essential to preserve biological robustness and clinical safety. Ultimately, adipose tissue should be recognized not merely as a convenient autologous source of regenerative cells but as a context-sensitive biologic product whose quality reflects the physiological and operational conditions under which it is obtained.

Bridging the elective–emergency divide is, therefore, not a procedural refinement—it is a necessary step toward ensuring reproducible, evidence-based integration of SVF-based regenerative strategies across all surgical settings.

## Data Availability

No new data were generated or analyzed in this study.
